# Closing the gaps in the HIV care continuum

**DOI:** 10.1371/journal.pmed.1002443

**Published:** 2017-11-21

**Authors:** Ruanne V. Barnabas, Connie Celum

**Affiliations:** 1 Departments of Global Health, Medicine, and Epidemiology, University of Washington, Seattle, Washington, United States of America; 2 Fred Hutchinson Cancer Research Center, Seattle, Washington, United States of America

## Abstract

In a Perspective, Ruanne Barnabas and Connie Celum discuss the implications of the accompanying Link4Health and Engage4Health studies for HIV care in sub-Saharan Africa

In July 2017, the Joint United Nations Programme on HIV and AIDS (UNAIDS) reported the effect of remarkable ongoing successes in the HIV care continuum: of the estimated 33 million HIV-positive persons worldwide, 70% know their status; among those people, 77% (19.5 million) are on antiretroviral therapy (ART) for HIV and 82% of persons on ART are virally suppressed, indicating successful treatment [1]. The goal set by UNAIDS for the HIV care continuum is to bring about testing of 90% of HIV-positive persons, to initiate ART among 90% of HIV-positive persons tested, and to achieve viral suppression among 90% of those on ART [2]. This 90-90-90 strategy is expected to achieve control of the HIV epidemic by 2030, with significant health and economic benefits among those treated and from the number of new HIV cases prevented. Although the UNAIDS goal appears to be within reach, innovative approaches are necessary to close the gaps and reach people not already in care in the many different affected countries, and to streamline services to achieve efficiency and preserve or improve quality of services.

In this issue of *PLOS Medicine*, two independent studies present notably consistent findings on the substantial impact of combination intervention strategies (CIS) on increasing linkage to and retention in the HIV continuum of care in two different settings in sub-Saharan Africa [3,4]. The evidence-based strategies to strengthen linkage and retention in HIV care in the CIS components included point-of-care CD4 testing, accelerated ART initiation, text message reminders, and noncash financial incentives to increase linkage and retention in HIV care.

Margaret McNairy and colleagues report on the Link4Health Study, a cluster-randomized controlled trial conducted in Swaziland that compared CIS (including noncash financial incentives) with the clinic standard of care (SOC) [3]. Batya Elul and colleagues report on the Engage4Health study, also a cluster-randomized controlled trial, which was conducted in Mozambique and compared SOC to CIS without financial incentives and CIS with financial incentives (CIS+) [4]. Both studies enrolled newly identified HIV-positive persons who were willing to initiate ART. The first step in the CIS was point-of-care CD4 testing to decrease the time to determination of ART eligibility, followed by a clinical screen to exclude criteria for delayed ART, counseling, and accelerated ART initiation. Once ART was initiated, both studies strengthened retention in care by providing reminders (phone calls or text reminders) and small noncash financial incentives (mobile phone vouchers, health educational packages, or travel reimbursements). The combined primary outcome was linkage to care at one month and retention at month 12, with secondary outcomes measuring the impact of CIS at each step in the HIV continuum. The Link4Health study randomized 2,197 HIV-positive persons to two arms and the Engage4Health study randomized 2,005 individuals to three arms. Both studies followed participants for 12 months with good ascertainment of outcomes through review of records and tracing participants who were lost to follow-up. At the time the studies were carried out in 2013–2015, prevailing national guidelines indicated initiation of ART at CD4 cell counts equal to or below 350 cells/mm3.

For the primary outcome of combined linkage within one month and retention at month 12, both studies found that CIS increased the effectiveness of linkage and retention by approximately 50% (Link4Health adjusted relative risk [RR] = 1.52, 95% confidence interval [CI]: 1.19–1.96; Engage4Health RR = 1.58, 95% CI 1.05–2.39). In addition, the Engage4Health Study found no additional impact of the noncash incentives, although notably the third study arm was smaller and might not have had sufficient power to see an effect of financial incentives. In the Link4Health Study, the impact of each intervention was not assessed separately.

## Differentiated service delivery to close gaps in the HIV care continuum

Both evaluations of the CIS interventions presented by McNairy and Elul and their respective colleagues are client-centered approaches that simplify and adapt services to meet people’s preferences and expectations while reducing unnecessary burdens on the health system—known as differentiated service delivery (DSD) [5,6]. DSD could facilitate achieving the 90-90-90 goals by reaching persons not currently in the health system and by maximizing health outcomes within constrained resources. Both the Engage4Health and Link4Health studies worked within public clinics demonstrating that CIS implementation and evaluation are feasible and acceptable, albeit with the additional resources provided through a research study. Nevertheless, these studies provide clear guidance on how clinics could streamline services and meet client needs. Specifically, reducing the number of steps to ART initiation remains relevant in the current era of offering ART to all people testing positive for HIV, irrespective of CD4 cell count. An example is the approach of Médecins Sans Frontières, in which adherence counseling is tailored to barriers identified for individual people, rather than providing comprehensive adherence counseling to all, with the benefit of reducing provider and patient time and streamlining the steps to ART initiation [7,8]. South Africa has recently provided guidance for ART initiation on the same day of HIV diagnosis for clinically stable patients, with people returning seven days later for review of baseline laboratory measurements and symptoms, further reducing delays to ART initiation [9].

## Measuring health impact is key for evaluation of DSD

DSD is an important focus area due to the challenges of strained health facilities being able to accommodate a rapidly growing number of HIV-positive persons entering HIV care. Many DSD strategies attempt to address the barriers of long waits and monthly medication refills and need to be robustly evaluated to measure their impact on health, costs, and scalability [10]. Indeed, not all DSD interventions are effective, such as the financial incentives in the Engage4Health Study, which was not shown to improve linkage and retention, although that comparison might have been underpowered. Notably, the Link4Health and the Engage4Health studies are implementation sciences studies which were conducted in public HIV programs in Swaziland and Mozambique, respectively, and encountered challenges of assessing outcomes with missing health records, incomplete electronic records, and limitations of missing data in terms of whether participants linked to care elsewhere, were no longer in care, or died. However, these limitations of implementation science research are balanced by increased generalizability and relevance to scalable delivery models. Future DSD studies would benefit from improved data capture and tracking, which are necessary to ascertain outcomes at both the individual and population levels.

## The next steps for CIS interventions

The Link4Health and Engage4Health studies represent the beginning of making HIV care streamlined and more client-centered. Changes in HIV treatment, which are likely to include longer-acting ART formulations [11,12] and integration of other chronic disease diagnosis and management into HIV care platforms [13,14], present opportunities for continued re-engineering of health systems. Building on the two studies presented in this issue and anticipating future changes, key priorities for future work include: first, reaching people who know their status but are not engaged in HIV care as they are missing from the HIV care continuum; and second, consistently and reliably reporting the impact on health, particularly viral suppression, and over longer durations than 12 months of follow-up. Routine evaluation of health outcomes requires robust clinical records and reporting that accounts for missing data, and in many cases, this will require strengthening data capture systems. Surveillance can play a key role in providing evidence for key gaps in routine data [15]. Lastly, operations research into new strategies also requires rigorous assessment and further work is needed on adaptive interventions [16], which will be essential as additional delivery options are developed for treatment of HIV-positive persons. Collectively, implementation and evaluation of innovative differentiated care models should bring us closer to the UNAIDS 90-90-90 goals.

## Abbreviations

ART: antiretroviral therapy
CI: confidence interval
CIS: combination intervention strategies
DSD: differentiated service delivery
RR: relative risk
SOC: standard of care
UNAIDS: Joint United Nations Programme on HIV and AIDS
